# Pellagra

**Published:** 1910-04

**Authors:** D. N. Sage

**Affiliations:** Atlanta, Ga.


### PELLAGRA.

BY D. N. SAGE, M. D., ATLANTA, GA.

In beginning this paper on Pellagra, I wish to state that I claim nothing in it of especial importance as original. Many experiments as to etiology and treatment of Pellagra have recently been made in this country and yet it is found that most of these practices have been used before in Europe where the disease has been common for several hundred years. Many papers have been read and many articles written, very largely exhibiting the same theories and the same results. In this paper I merely hope to mention the facts and theories which seem to me to be most in accord with the disease itself and which seem to be most generally accepted.

If I use some prominent observer's findings or apt expressions I have already warned you of that fact and take this occasion to give credit to the many able men who have spent much time and labor studying this disease. My personal experience has merely consisted in an observation of the conduct of five private cases and of eleven hospital cases some of which I was not able to follow long.

Pallagra was first diagnosed as a new disease in 1735 by physicians in Spain. In 1750 it appeared in Italy. At about this time Indian corn was first being shipped in large quantities to Spain and Italy. But it was not until 1810 that mouldy grain or maize was connected with Pellagra. In 1821 Indian corn was conceded to be the most important if not the sole medium through which the disease has origin. Since these years these countries, and more especially Roumania, have continually had thousands of cases, epidemics seeming to follow the importation of Indian corn and especially where the corn was found to have been poorly cured and poorly preserved.

In 1902 the first cases in the United States were reported. Since that year the south and east have reported a rapidly increasing number of pellagrins.

*Etiology*:

As to the cause of Pellegra there is still much dissention, all owing to the fact that no specific organism or toxin has as yet been satisfactorily demonstrated. Numerous moulds and fungi have been carefully studied by all the known methods, the results being generally unsatisfactory. The chemical theory has retained some supporters as has also the toxic theory. But each in its turn has failed to be demonstrable.

We know that the disease has followed in the wake of poorly preserved corn. We know that epidemics cease upon regulating the quality of corn used in the country.

At this date there seems to be very few who doubt that Indian corn is the most important if not the sole medium through which the disease attacks mankind. Furthermore, it seems more than likely that either the toxic or specific theory will prevail. Some even now claim to have isolated the active cause.

As predisposing causes the most common are alcoholism, mental depression, abject poverty and its usual environments, poor quality and quantity of food, lowered resistance or loss of vitality from any cause, such as an acute or chronic illness. Certain it is that a diet composed largely of corn and its products is predisposing.

Good corn is readily admitted to be a very excellent food, but it is doubtful when it is good. In one season I saw some hundred cars of hot and spoiling corn aggregating many thousand bushels dried and sold to be ground into meal or made into whiskey which again was largely used by the people of Georgia.

In that year many million bushels were similarly handled in the southern States. Some of that meal was mixed into all manner of fancy foods, some sold in flour mixtures, and much as first or second class meal. We never know into what dish the corn product is mixed or whether it is good or bad. The good is mixed with the bad and the mixture sold as first-class.

*Pathology*:

The morbid anatomy is rather slight considering the severity of the disease. The principal changes are found in the nervous centers. These may begin early or late and are not positively determined except by a post mortem and the microscope. They are only slightly marked in some cases.

The intestinal tract may have a few small ulcers really local inflammatory foci. Intersusseptions are common, possibly the result of loss of glandular activity. In one case which came under my observation the buchal mucous membrane was raw and waxy and the perineum was worse, the skin and membranes being practically destroyed.

*History*:

Usually the history of one or more severe attacks of diarrhea, each attack lasting from five days to several weeks and producing from five to fifteen or twenty copious watery stools in twenty-four hours, may be elicited. The patient probably thought some error in diet had been responsible, but will tell you that ordinary methods in use did not materially check this diarrhea. These attacks may have occurred at intervals covering the last several years or the patient may then be suffering from the first.

Nausea and pain may accompany the diarrhea or may be recalled as common for a number of years or as quite recent.

The stools may occasionally contain blood, but this is often found as secondary to hemorrhoids and not from lesions from above the rectum.

*Nervous*:

Perhaps before the patient consults the physician he or his family have noticed a change in disposition. The patient is usually nervous and irritable or may become melancholly and apathetic.

Probably insomnia will be a marked symptom, the patient often sleeping only two to four hours regularly in the twenty-four.

Later the family tell you that the patient acts and talks queerly. This may not get worse or may not occur, some dying without these symptoms and others becoming entirely insane.

*Skin*:

Usually as a rather late change comes the dermatitis, affecting the back of the hands and wrist, the feet and ankles, the face and chest. This dermatitis varies greatly from a dry, non-inflammatory scaling, to a moist, very red, scaly process, usually most prominent on the hands, and often not noticed elsewhere. The tongue is variable, but often presents the fiery red of sprue or may be deeply serrated.

The liver is thought by most to be little affected.

The urine is generally about normal with an occasional trace of albumen and a low specific gravity.

One or both pupils may be dilated and the conjunctiva may be inflamed and thickened to such an extent that the lachrymal ducts do not satisfactorily afford passage for the gummy secretions.

Stooping shoulders and loss of weight and strength may set in early or late. Some seem to be well nourished up to the very last, but usually the tissue is fat and flabby and the prognosis probably worse.

The pulse is often full and almost normal, though haemio murmurs are common in the later stages. There is little temperature as a rule. A constant fever is a grav esymptom and a very high temperature is usually a danger signal that the end is near.

*Diagnosis*:

In some cases the dermatitis is absent or so slight as to be overlooked, but those cases are rare indeed. When present it is very suggestive despite the fact that it is usually late.

The absence of a syphilitic history or the anti-syphilitic treatment will clear that point. The miscroscope will usually show the hook worms if present or a proper vermicide will cause evacuation of the worms.

Tuberculosis also has its bacillus and better still will be found the tuberculin reaction test.

Sprue gives large quantities of mucous in the stools and readily yields to tannic acid.

Leprosy has some symptoms in common, but the early intermittent fever or the bacillus either would serve to show the distinction.

*Prognosis*:

The prognosis is always grave. This may partly be accounted for by the fact that most diagnoses are made from the external symptoms which usually appear late and are secondary to the neuroses.

In Europe the mortality per year is now placed from five, thirteen to fifty per cent. Their statistics do not show how many of their cases eventually have other attacks of which they die.

The prognosis is better in non-asylum cases, probably owing to environments, etc.

The best prognosis is to be made in cases of several years' standing in which there is little weakening and no mental symptoms. The worst is the acute type with mental symptoms marked. The second acute attack in the same season is usually more fatal than a single attack.

The complications play an important part in the prognosis. Among the most important and frequently found are malaria, intestinal parasites, nephritis, bronchitis, pneumonia, decubitus gangrene, tuberculosis, and child-birth.

A subsultus or tremor, head reaction, progressive emaciation and diarrhea often point to the end.

*Treatment*:

The treatment resolves itself into prophylactic and medicinal of which the former is conceded to be by far the most beneficial.

Through prophylaxis we attempt to remove all predisposing causes and thereby aid the entire community. A liberal diet and an excess of red meats is indicated.

Baths and douches are good when not annoying to the patient.

Warn the patient to avoid the sun in summer and you may prevent marked dermatitis. The use of gloves or bandages may serve this purpose and yet not keep the patient in-doors. With this one reminder the prophylaxis is almost identally the same as for tuberculosis.

Rest in bed is essential in acute attacks. A change to colder climate is often beneficial.

The medicinal agents employed are as yet non-specific and from the nature of the disease it would appear that, as in tuberculosis, the greater damage usually being accomplished before diagnosis, a specific might save life and yet leave the patient in the insane asylum.

The treatment depends largely upon the use of arsenic in one of its various compounds as the systematic and the ordinary symptomatic treatment. Sodium chloride is sometimes being used for its anti-toxic effect.

Of the arsenic, Fowler's solution three to thirty min. may be given daily, with an intermission of a few days to a week from time to time.

Among the newer compounds atoxyl is receiving a great deal of attention. Many think it the best form in which to give arsenic. It is employed in doses from one-third to three grains hyperdermically, every other day. The arsenic is liberated very slowly and more continuously with less danger of toxic effects. However, toxic symptoms must be carefully watched for the dose being very gradually increased in each of the arsenic preparations. As much as ten grains of Atoxyl have been given in one day to patients to whom the drug has long been administered and get no bad results. While others will not stand half as much, some cases of blindness being reported as the result of small quantities of arsenic.

Insomnia will require one or other of the hypnotic ointments, such as icthyol and lanolin, iodin, boric acid, one per cent. picric acid aqueous solution, or other preparations may be required for the dermatitis. Calomel and bismuth and opium may be used for the diorrhoea.

Pain is not a frequent symptom, but morphine is commonly used if pain is present.

*Discussion of Dr. Sage's paper.*

Dr. C. M. Niles, Atlanta, Ga., led the discussion. One point I want to bring out in the discussion. We have to admit that Sprue and Pallagra are related, it is hard to differentiate between them. I want to call your attention to the fact that in Sprue you get the spots on the tongue, not always in Pellagra. Also that in Pellagra the diarrhea is intermittent; in Sprue it is constant, with a great deal of gas. One of the main points that we get in Pellagra is the marked brain features, which in Sprue we do not get.

I know of a case, a man, ordinarily he was very fond of his family of children, but after becoming affected with Pellagra he grew to be nervous, depressed, and very cross with the children, showing decided trouble with his brain. Pellagra is a live wire in this country, cropping out in the entire country among the rich and poor.

Dr. Thos. F. Taylor, Tuskegee, Ala.—One reason I came to this meeting was that I wanted to hear something more of Pellagra. It has been my misfortune to see 50 or 70 cases of Pellagra before the diagnosis was made. That was in the Insane Hospital at Tuscaloosa, Ala. Dr. Sage has covered all the facts we know up to date; the general symptons have been enumerated.

These cases we had in the hospital we called auto-intoxication, which it is, with skin symptoms, but we did not always have the skin symptoms. One point is the marked salivation, and the peculiar odor. The patient with a red tongue, in acute cases, spits off quantities of foul smelling saliva which is peculiar to the diseases.

As to the treatment, they found after taking the patients off corn meal, the number of cases diminished. After examination much of the corn had been found unfit for use. As to corn meal, we as doctors are robbed of a great deal of our material as we are now afraid to give bread pills. It has been my experience that 80 or more per cent. of the acute cases die. Since I have been in private work, have had only two cases. One acute where diognosis was made after death. The other case was entirely chronic, the only symptoms I got was the eruption on the back of hands, and the callous tongue, with entire absence of nervous symptoms, very slick, glazed appearance of the tongue with red tip. Eruption had begun to fade. Treated with arsenic, 10 to 15 drops of Fowler solution. She has recovered so far, but this is the fifth summer she has had the trouble.

The doctor did not introduce the transfusion method of treatment. Recent reports from the hospital show they have used the transfusion method; took blood from one patient and transferred to another, and patient recovered, but do not know whether it has occurred since then.

Dr. J. H. McDuffie, Columbus, Ga.—I have seen three cases in the past few years, one of which I think died with tuberculosis of the skin and bowels, but this has been touched upon.

I have seen about twenty-five cases, all have had dermititis, from a slight eruption to a solid sore covering all affecetd parts. All cases I have seen have died. In regard to the change in disposition, this has aided in my diagnosis of two cases, I have given these cases no special treatment. Have used iron and arsenic, and dieted them with milk and eggs, but the failure of these getting well led me to give others more liberal diet. I have one case now under treatment since last May, whose sister died with this trouble last year.

This lady has had two attacks in the two preceding springs, though not bad attacks. I put her on arsenic, and gave a diet of milk and eggs, and told her what was the matter. Finally she went to Atlanta to consult a physician there, as I suppose she did not consider that I was giving her the treatment she ought to have. I do not know her success up there, but she finally returned to me, and I kept her at Chalybeate Springs during the summer, now her skin has cleared up, and she seems to be in fairly good condition of health. I recently put her on a preparation of arsenic and iron, one tablet increased to four tablets every other day, but what the spring will do for her I cannot say.

Dr. J. M. Poer, West Point, Ga.—One fact not touched upon is that we are dealing with chronic, not acute disease. The patient probably having been affected ten or eleven years, as the Doctor says, usually in the Spring. I have seen several of these cases who are very much improved, but cannot say what the Spring will bring forth. I want to stress the importance of early diagnosis of Pellagra. It must be diagnosed in that beginning stage of diarrhea, acidity of stomach, and other disturbances, as then with proper diet, etc., these patients could be cured, though I do not claim to know how to do this myself.

Dr. D. Y. Sage, in closing.—I notice that it has been pronounced contagious. It has been my experience that where a whole family have it, it is from the same cause. On investigation it is found that in a family of nine, the only one who did not have it never used corn meal. Iron has been used where there is great enemia, where it is beneficial so long as the enemia exists, after that the damage done to the stomach is great.

As to the transfusion method I did not touch on that, as I was afraid of it, but hope it will be of great aid in the future. Think the patient must be infused from one who has himself recovered from Pellagra.

